# Unmasking early colorectal cancer clues: in silico and in vitro investigation of downregulated IGF2, SOCS1, MLH1, and CACNA1G in SSA polyps

**DOI:** 10.1007/s11033-024-09683-3

**Published:** 2024-06-14

**Authors:** Seyedeh Nasim Mirbahari, Nayeralsadat Fatemi, Sanaz Savabkar, Vahid Chaleshi, Neda Zali, Mohammad Yaghoob Taleghani, Ebrahim Mirzaei, Leili Rejali, Pardis Ketabi Moghadam, Ehsan Nazemalhosseini Mojarad

**Affiliations:** 1 Faculty of Sciences and Advanced Technologies in Biology, University of Science and Culture, ACECR, Tehran, Iran; 2 Department of Genetics, Reproductive Biomedicine Research Center, Royan Institute for Reproductive Biomedicine, ACECR, Tehran, Iran; 3 Basic and Molecular Epidemiology of Gastrointestinal Disorders Research Center, Research Institute for Gastroenterology and Liver Diseases, Shahid Beheshti University of Medical Sciences, Tehran, Iran; 4 Department of Medical Genetics, School of Medicine, Iran University of Medical Sciences, Tehran, Iran; 5 Gastroenterology and Liver Diseases Research Center, Research Institute for Gastroenterology and Liver Diseases, Shahid Beheshti University of Medical Sciences, P. O. Box: 1985717413, Tehran, Iran; 6 Department of Surgery, Leiden University Medical Center, P.O. Box 2333 ZA Leiden, Netherlands

**Keywords:** Colorectal cancer, Sessile serrated adenoma polyp, Weissenberg panel, Hyper methylation

## Abstract

**Background and aim:**

Colorectal cancer (CRC) originates from pre-existing polyps in the colon. The development of different subtypes of CRC is influenced by various genetic and epigenetic characteristics. CpG island methylator phenotype (CIMP) is found in about 15–20% of sporadic CRCs and is associated with hypermethylation of certain gene promoters. This study aims to find prognostic genes and compare their expression and methylation status as potential biomarkers in patients with serrated sessile adenomas/polyps (SSAP) and CRC, in order to evaluate which, one is a better predictor of disease.

**Method:**

This study employed a multi-phase approach to investigate genes associated with CRC and SSAP. Initially, two gene expression datasets were analyzed using R and Limma package to identify differentially expressed genes (DEGs). Venn diagram analysis further refined the selection, revealing four genes from the Weissenberg panel with significant changes. These genes, underwent thorough in silico evaluations. Once confirmed, they proceeded to wet lab experimentation, focusing on expression and methylation status. This comprehensive methodology ensured a robust examination of the genes involved in CRC and SSAP.

**Result:**

This study identified cancer-specific genes, with 8,351 and 1,769 genes specifically down-regulated in SSAP and CRC tissues, respectively. The down-regulated genes were associated with cell adhesion, negative regulation of cell proliferation, and drug response. Four highly downregulated genes in the Weissenberg panel, including *CACNA1G*, *IGF2*, *MLH1*, and *SOCS1*. In vitro analysis showed that they are hypermethylated in both SSAP and CRC samples while their expressions decreased only in CRC samples.

**Conclusion:**

This suggests that the decrease in gene expression could help determine whether a polyp will become cancerous. Using both methylation status and gene expression status of genes in the Weissenberg panel in prognostic tests may lead to better prognoses for patients.

## Introduction

According to the National Cancer Institute, in 2021, an estimated 1,486,920 people were living with colorectal cancer (CRC) in the United States [1]. It is generally well-known that CRC arises from pre-existing colonic polyps [2]. Premalignant epithelial polyps of the large intestine comprise several different subtypes, such as adenomatous and sessile serrated adenoma/polyp (SSAP), that are capable of development of different types of colorectal carcinomas due to their different genetic and epigenetic characteristics [3]. Three principal pathways which have been associated with these characteristics are chromosomal instability (CIN), microsatellite instability (MSI), and CpG island methylator phenotype (CIMP) pathway [4]. The latter is found in 15–20% of sporadic CRCs and it occurs when DNA methyltransferases (DNMTs) promote hypermethylation of promoter-associated CpG-rich regions of tumor suppressor and DNA mismatch repair genes such as *p16*, *PTEN*, and *MLH1*, leading to development and progression of CRC [5]. Various studies have indicated an association of CIMP with clinical, histopathological, and epidemiological characteristics, such as older age, female sex, tumor location in the proximal colon, poorly differentiated tumors, and high rates of *BRAF* and *TP53* mutations [6–10]. Thus, the hypermethylation phenotype of certain gene promoters can be served as a biomarker for the early detection of CRC.

Detecting CRC early poses significant challenges as it often progresses asymptomatically in its initial stages, complicating timely diagnosis [11]. Moreover, CRC encompasses diverse molecular subtypes, contributing to the complexity of accurate diagnosis and prognosis determination [12]. Treatment of CRC encounters obstacles such as chemotherapy resistance, underscoring the need for more effective therapeutic strategies [13]. Identifying individuals at high risk of developing CRC remains crucial for early intervention and prevention [14]. Given these challenges, the study’s objectives of identifying prognostic genes in CRC and SSAP hold immense significance. By shedding light on potential biomarkers for improved diagnosis and treatment outcomes, the study aims to address critical gaps in current CRC management strategies [15]. SSAP, characterized by their serrated appearance and typically flat or sessile morphology, have garnered increasing attention in cancer research due to their potential role as precursor lesions for CRC [16]. Despite their relatively benign appearance, SSAPs have been associated with an increased risk of developing CRC, particularly in cases where these lesions are left undetected or inadequately removed during screening. Detecting SSAPs presents unique challenges compared to other types of colorectal polyps, as they may be subtle and easily overlooked during endoscopic examinations. Moreover, the sessile nature of these lesions can complicate their complete resection, further contributing to the risk of CRC development [17]. Therefore, improving methods for the detection and surveillance of SSAPs is imperative for effective CRC prevention strategies. While advanced imaging techniques such as chromoendoscopy and narrow-band imaging (NBI) have shown promise in enhancing SSAP detection rates, their widespread adoption and efficacy in routine clinical practice require further validation [18]. Additionally, molecular markers, including DNA methylation patterns, have emerged as potential tools for identifying high-risk SSAPs that may progress to CRC [19]. Understanding the clinical significance of SSAPs and addressing the challenges associated with their detection and management are crucial steps toward improving CRC prevention and early intervention efforts. Recent advances in high-throughput technologies have facilitated the establishment of DNA methylation panels to determine the CIMP status of specific genes in CRC tumors [20, 21]. In 2006, Weisenberger et al. introduced a quantified CIMP panel, which serves as a collection of genes widely employed as biomarkers in DNA methylation studies within cancer research. The Weissenberg panel encompasses 1505 CpG sites situated in the promoter regions of 807 genes associated with cancer. These genes were carefully selected due to their significance in cancer biology and their potential usefulness as targets for diagnosis and treatment purposes [22]. Some of the genes included in the Weissenberg panel are *APC* (Adenomatous polyposis coli), *BRCA1* (Breast cancer 1), *CDH1* (Cadherin 1), *CDKN2A* (Cyclin-dependent kinase inhibitor 2 A), *MLH1* (MutL homolog 1), *PTEN* (Phosphatase and tensin homolog), *TP53* (Tumor protein p53) and *VHL* (Von Hippel-Lindau tumor suppressor) [22, 23]. The Weissenberg panel was chosen based on its established significance in previous research related to CRC and SSAP. This panel comprises genes that have been consistently implicated in CRC pathogenesis and progression, making it a valuable tool for identifying potential prognostic markers. Additionally, the identification of differentially methylated genes can provide insights into the molecular mechanisms underlying cancer development and progression and may lead to the development of new treatments for cancer. Despite the various panels to assess CIMP status, the panel introduced by Weisenberger has been reviewed and validated in several studies [22, 24–26]. As far as we know, there is no correlation study between the methylation status of this panel and the expression of them at the time of polyp initiation and the spread of CRC; therefore, in the present study, we aimed to identify genes that are differentially expressed in SSAP and CRC using bioinformatics analysis. By utilizing previous studies and matching our classification with known panels, we successfully identified some common effective genes. In the next step, we have selected this final group of genes to be tested in wet lab phase.

## Methods

As it is shown in Fig. 2, in the in silico phase of the study, genes involved in the pathways of interest are identified, as are genes that are not involved in these pathways. Genes that are involved in the pathways and are differentially expressed or methylated in the cells of interest are then identified. Finally, pathway analysis tools are used to identify the GO terms and pathways associated with these genes. In the in vitro phase of the study, the methylation pattern and protein-protein interaction of the genes identified in the in silico phase are evaluated. The effect of these genes on the survival rate of the cells of interest is also assessed.

### In-silico analysis

The microarray data were retrieved from NCBI’s Gene Expression Omnibus database (http://www.ncbi.nlm.nih.gov/geo), with GEO accession numbers GSE7946 and GSE198692. GSE7946 is composed of 22 SSAP samples and 22 adjacent non-tumor mucosa samples from the same patients. GSE198692 is composed of 7 SSAP samples and 4 normal samples. All GSE series matrix files set were downloaded and parsed by the GEO query package [27] using R version 3.6.0, and the GSE series values were normalized with R. The linear models for microarray data (Limma) package [28] were employed to determine Differentially Expressed Genes (DEGs), which were significantly upregulated and downregulated in SSAP. DEGs were selected based on univariate tests according to the q-value (q < 0.05) and log2 fold change (log2 FC ≥ 2).

#### Identification of differentially expressed genes in CRC

The Gene Expression Profiling Interactive Analysis (GEPIA) database (http://gepia.cancer-pku.cn/), which is a web-based tool that provides fast and customizable functionalities based on TCGA and GTEx data, was used to compare gene expression between CRC and normal tissue. Differential expression analysis was conducted using default parameters, with statistical significance determined based on a p-value threshold of 0.05 and log2 fold change (|log2 FC| ≥ 1).

#### Identifying differentially expressed genes and specificity in CRC and SSAP through Venn diagram analysis

In this phase, we constructed Venn diagrams to identify genes that were specific to SSAP, specific to CRC, and genes that were common to both upregulated and downregulated genes. The parameters used for gene selection included statistical significance (q-value < 0.05) and a log2 fold change threshold of ≥ 2.

#### Gene ontology and pathway enrichment analysis

Gene ontology analysis of the biological processes (BP), molecular function (MF), and cellular component [6], as well as the Kyoto Encyclopedia of Genes and Genomes (KEGG) pathway enrichment for the DEGs specific to CRC, were conducted using the Database for Annotation Visualization and Integrated Discovery (DAVID; https://david.ncifcrf.gov). The statistical significance threshold was set at a p-value less than 0.05.

#### PPI network of Weissenberg panel

The protein-protein interactions (PPI) of IGF2, SOCS1, MLH1, and CACNA1G genes were analyzed using the STRING database (https://string-db.org/).The confidence score threshold for defining protein-protein interactions was set at 0.7. To visualize the interactions of the proteins encoded by these four genes with other proteins, the Cytoscape software (http://www.cytoscape.org/) was used.

#### Overall survival analysis

To evaluate the prognostic value of the panel genes, an analysis was performed using the Kaplan-Meier plotter. Patient data from the GEPIA database were utilized for this analysis [11]. The survival plots, gene expression levels, and stage plots were generated to investigate the relationship between the selected genes and common cancers. The statistical significance threshold for survival analysis was set at a p-value less than 0.05.

#### Evaluation of methylation and expression pattern of genes

After obtaining the methylation results, *IGF2*, *SOCS1*, *MLH1*, and *CACNA1G* were entered into the UALCAN database (http://ualcan.path.uab.edu) for further studies. The UALCAN database was used to examine the promotor methylation levels and expression of *these* genes with regard to clinicopathological features in CRC patients. This analysis utilized default parameters provided by the UALCAN platform, with statistical significance determined based on a p-value threshold of 0.05 [29].

### In-vitro analysis

**Fig. 1:**
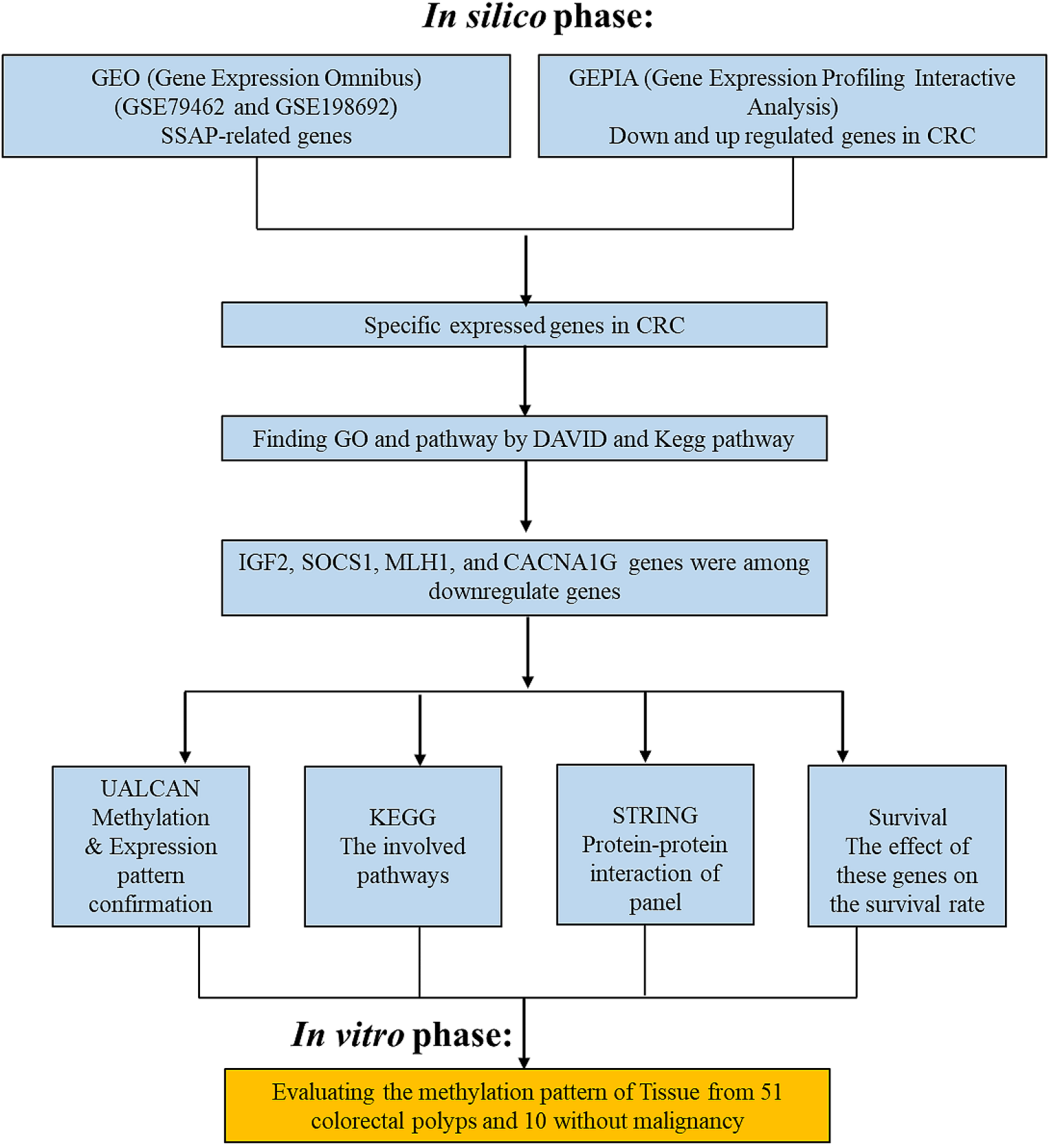
Flow diagram of a study to investigate the role of SSAP-related genes in colorectal cancer (CRC). This study investigated the role of SSAP-related genes in colorectal cancer (CRC). In the in silico phase, gene expression data of SSAP-related genes from two GEO datasets (GSE79462 and GSE198692) were analyzed to identify downregulated and upregulated genes in CRC. Four downregulated genes (IGF2, SOCS1, MLH1, and CACNAIG) were selected for further analysis. In the in vitro phase, the methylation pattern of the four selected genes was evaluated in tissue samples from 51 colorectal polyps and ten normal samples. The effect of the four selected genes on survival rate was also analyzed. The study found that the four downregulated genes may play a role in the development and progression of CRC. Further studies are needed to validate these findings and to investigate the mechanisms by which these genes contribute to CRC

After conducting computer-based studies, we identified four genes including (*IGF2*, *SOCS1*, *MLH1*, and *CACNA1G*) with significantly decreased expression in the bioinformatics analysis, which was part of the Weissenberg panel. To confirm our findings, we further studied the expression and methylation status of these genes. Figure 1 indicates the workflow of our study. Between 2018 and 2021, fresh frozen tissue samples were obtained from various study groups at Taleghani Hospital, affiliated with Shahid Beheshti University of medical sciences in Tehran, Iran. These groups included invasive adenocarcinoma (*n* = 14), SSAP (*n* = 9), and control cases without malignancy (*n* = 34). Patients who had undergone radiotherapy or chemotherapy were excluded from the study. It is noteworthy that the study received approval from the Medical Ethical Committee of SBUMS and informed consent was obtained from all participants.

#### RNA and DNA extraction

Total RNA was extracted from the colonic tissues using the Trizol® kit (ThermoFisher Scientific, United States) according to the manufacturer’s protocol. 50 to 100 mg of the same tissues were used to isolate DNA using the QIAamp DNA mini kit (Qiagen, German), following the manufacturer’s instructions. The quantity and integrity of the extracted RNA and DNA were meticulously evaluated using Thermo Scientific NanoDrop 1000 and agarose gel electrophoresis, respectively, as critical quality assurance measures to validate sample integrity and data reliability. These quality control steps are essential for minimizing experimental variability and ensuring the validity of downstream analyses.

#### Real-time quantitative PCR

The PrimeScript cDNA Synthesis Kit (Takara, China) was used to synthesize cDNA following the manufacturer’s instructions. Real-time PCR was performed on an Applied Biosystems 7500 Real-Time PCR System using an SYBR Green Real-Time PCR Master Mix (Takara, China), adhering to the manufacturer’s recommendations to maintain assay standardization. The specific primers used for gene expression quantification are listed in Table 1. Thermal cycling conditions were as follows: an initial denaturation step at 95 °C for 30 s, followed by 40 cycles of denaturation at 95 °C for 5 s, annealing at 58 °C for 34 s, and extension at 72 °C for 34 s, with a final extension step at 72 °C for 15 s. The relative expression of the selected genes was determined by normalizing to β-actin via the 2^−ΔΔCt^ method, incorporating robust data normalization and interpretation techniques for reliable results.

**Table 1:** Specific primers used in MSP and Real-time PCR, and sizes of the PCR products

Gene	Annealing Temperature (ºC)	Size (bp)	Methyl/Un-methyl	Sequence
MLH1	60	130	F Methyl	5′-AGGAAGAGCGGATAGCGATTT-3′
			F Unmethyl	5´- CCTCCAGGATGTATTTCACCCAG-3´
			R Methyl	5′-TCTTCGTCCCTCCCTAAAACG-3′
			R Unmethyl	5´- ACGGACCATCTGGTAAGCGTAG-3´
		214	Forward	5´- AAGGAAATGACTGCAGCTTGTAC-3′
			Revers	5´- GTTCTTCACTAAGCTTGGTGGTG-3′
CACNA1G	60	139	F Methyl	5´- TTTTTTTTGAGGGGCGTC − 3´
			F Unmethyl	5´- GATATTTTTTTTGAGGGGTGTT − 3´
			R Methyl	5´- CGACAAATCGTTAAACCG − 3´
			R Unmethyl	5´- CCCAACAAATCATTAAACCA − 3´
		150	Forward	5´- TCACGCAGCTCAACGACCTGTCC-3´
			Revers	5´- GGCTGTCCTGGCTCAAGTAGAAG − 3´
SOCS1	60	150	F Methyl	5′- TTG TTC GGA GGT GGA TTT -3′
			F Unmethyl	5′- ACAAGCTGCTACAACCAGGG-3′
			R Methyl	5′-TTA TGA GTA TTT GTG TGT ATT TTT -3′
			R Unmethyl	5′-ACTTCTGGCTGGAGACCTCA-3′
		81	Forward	5′-TTTTCGCCCTTAGCGTGAAG-3′
			Revers	5′-CATCCAGGTGAAAGCGGC-3′
IGF2	60	111	F Methyl	5´- GTAGTTCGGTTCGTAGGTCG − 3´
			F Unmethyl	5´- TAGGTAGTTTGGTTTGTAGGTTG − 3´
			R Methyl	5´- CGATAACGACACCGAAACCG − 3´
			R Unmethyl	5´- CAATAACAACACCAAAACCACTC-3´
		214	Forward	5´- GGGCAAGTTCTTCCAATATGA-3´
			Revers	5´- TCACTTCCGATTGCTGGC-3´
B actin	60	161	Forward	5´- GAGACCTTCAACACCCCAGCC-3´
			Revers	5´- AGACGCAGGATGGCATGGG-3´

#### Bisulfite modification

Isolated DNA was modified by sodium bisulfite using an Epitect bisulfite kit (Qiagen, Germany) according to the manufacturer’s recommendations. For methylation-positive and negative controls, human HCT116 DKO N samples (ZRC175286 and ZRC174904, respectively) were obtained from ZYMO Research, USA, and utilized as controls to verify the effectiveness of the bisulfite modification process and to validate the specificity of the methylation-specific PCR assay. These controls play a crucial role in confirming the reliability and reproducibility of the methylation analysis, providing confidence in the obtained results.

#### Methylation-specific PCR

The bisulfite-modified DNA was subjected to a methylation-specific polymerase chain reaction (MSP) for four CIMP markers (*IGF2*, *SOCS1*, *MLH1*, and *CACNA1G*). Methylated (mMSP) and unmethylated (uMSP) primers were designed using Primer3 software (version 0.4.0), and the sequence details are provided in Table 1. PCR reactions were carried out in a total volume of 12.5 µL, comprising 1.25 µL 5XPCR buffer, 0.25 µL dNTP-mix (1 mmol/L of each), 0.25 µL of magnesium chloride (50 mmol/L), 0.75 mL of each primer (20 pmol/mL), 0.125 µL of HotStart Taq DNA polymerase (2.5 units/µL, Biolabs, USA), and 200 ng of bisulfite-modified DNA. The thermal cycling program consisted of an initial denaturation step at 95 °C for 10 min, followed by 40 cycles of denaturation at 95 °C for 15 s, annealing at 60 °C for 30 s, and extension at 72 °C for 15 s, with a final extension step at 72 °C for 10 min. These rigorous experimental conditions and controls were implemented to minimize variability and ensure the accuracy of the methylation-specific PCR results. The reactions were performed on the 7900HT Sequence Detection System version 2.3 (SDS2.3; Life Technologies, USA).

#### ROC curve

The overall diagnostic performance of methylation test was evaluated using a ROC curve generated by (http://www.graphpad.com/quickcalcs/ConfInterval1.cfm (accessed November 2015). The ROC curves plot the true positive rate (sensitivity) against the false positive rate (1-specificity) at different threshold values of a diagnostic test. The area under the ROC curve [30] is a measure of the diagnostic accuracy of the test, with a value of 1 indicating perfect discrimination between cases and controls, and a value of 0.5 indicating no discrimination beyond chance.

### Statistical analysis

To identify DEGs, the multiple t-test was used and the Benjamini and Hochberg method was employed to adjust the q-values [31]. The difference in survival curves was compared using the log-rank test. The predictive performance of the genes was determined by calculating the area under the ROC curve [30]. The functional categories for enrichment analysis were downloaded from KEGG and the hypergeometric distribution model was used to test whether a set of observed genes in a functional term was significantly greater than what was expected by random chance.

## Result

### Gene ontology of cancer-specific genes

**Fig. 2:**
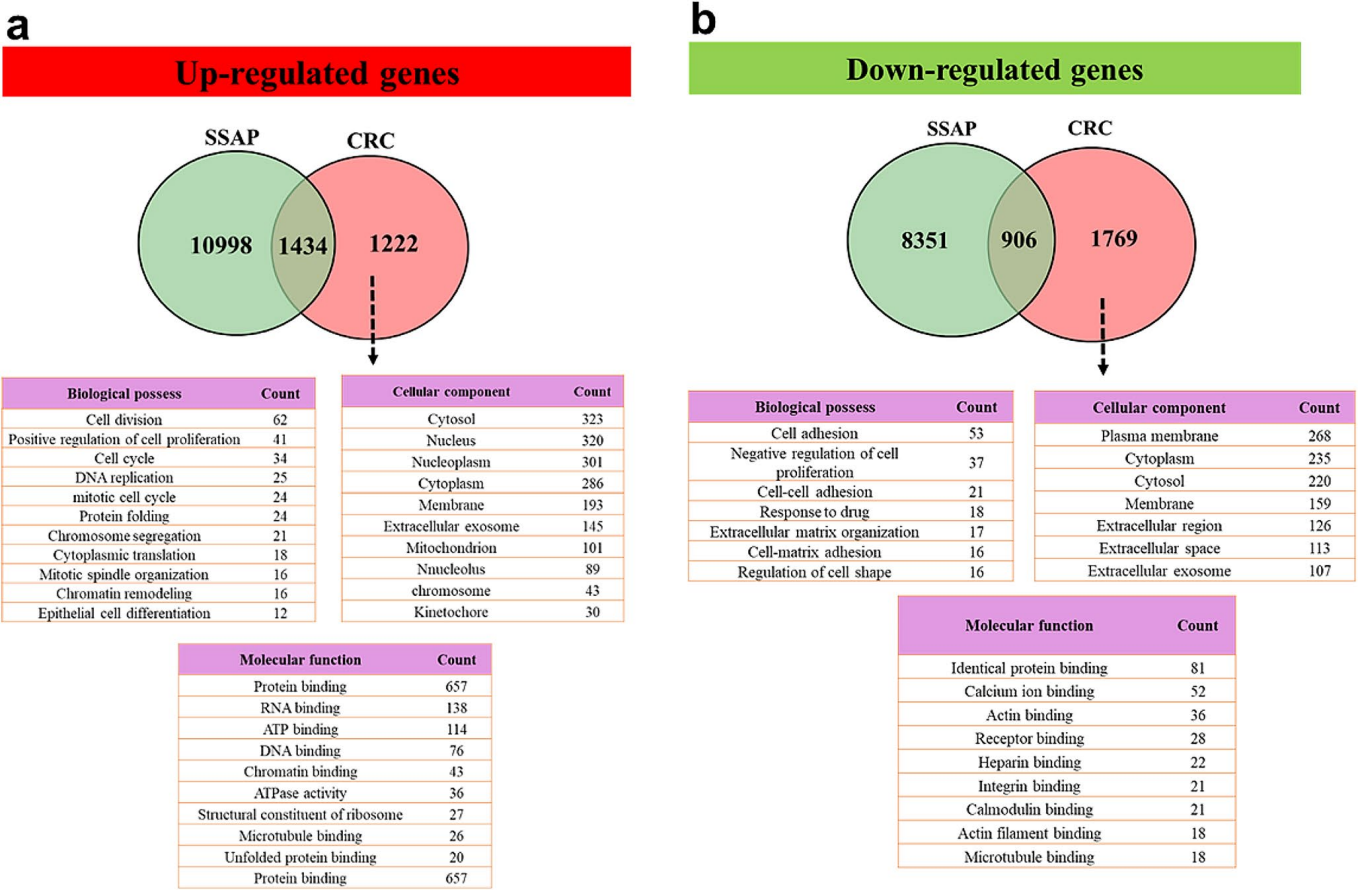
Venn diagram showing the relationship between up-regulated and down-regulated genes in colorectal cancer (CRC). It shows the overlap between up-regulated and down-regulated genes in CRC compared to normal tissue. There are 10,998 up-regulated genes and 1434 down-regulated genes in CRC. 323 genes are both up-regulated and down-regulated in CRC. It also also shows the overlap between up-regulated and down-regulated genes in CRC that are involved in specific biological processes. For example, there are 301 genes involved in cell adhesion that are up-regulated in CRC and 215 genes involved in cell adhesion that are down-regulated in CRC. This suggests that cell adhesion is an important biological process that is dysregulated in CRC. The Venn diagram also shows the overlap between up-regulated and down-regulated genes in CRC that are involved in specific signaling pathways. For example, there are 21 genes involved in the Wnt signaling pathway that are up-regulated in CRC and 21 genes involved in the Wnt signaling pathway that are down-regulated in CRC. This suggests that the Wnt signaling pathway is another important signaling pathway that is dysregulated in CRC

Regarding down-regulated genes, 8,351 and 1,769 genes were specifically down-regulated in SSAP and CRC tissues, respectively. Additionally, 906 genes were common in both types of tissues (Fig. 2, a). The GO of the down-regulated genes specific to CRC was examined and observed that these genes were predominantly linked to functions such as cell adhesion, negative regulation of cell proliferation, cell-cell adhesion, and drug response. Moreover, the most significantly enriched GO terms on cellular components (CC) for the down-regulated genes were the plasma membrane, cytoplasm, cytosol, membrane, and extracellular region. These genes were particularly enriched in molecular functions, including identical protein binding, calcium ion binding, actin binding, receptor binding, and heparin-binding (Fig. 2, b) As shown in Fig. 2, among the up-regulated genes, 10,998 were specifically expressed in SSAP, 1,434 genes were common in both samples and 1,222 genes were CRC-specific. Biological processes of positive regulation of cell proliferation, cell cycle, DNA replication, and mitotic cell cycle, and molecular functions such as protein binding, RNA binding, ATP binding, DNA binding, and chromatin binding were confirmed about up-regulated genes (Fig. 2, a).

### Four highly down-regulated genes were among the Weissenberg panel

Among cancer-specific down-regulated genes, a subset of 5 genes, have been found that were particularly important. *CACNA1G* (Calcium Voltage-Gated Channel Subunit Alpha1 G) is a gene that encodes a subunit of a voltage-gated calcium channel that plays a role in regulating calcium influx into cells [32]. *IGF2* (Insulin-like Growth Factor 2) is a gene that encodes a protein involved in cell growth and differentiation [33]. *SOCS1* (Suppressor of Cytokine Signaling 1) is a gene that encodes a protein that negatively regulates cytokine signaling pathways [34]. The identification of these 4 important genes within the Weisenberger panel suggests that they may play a critical role in the molecular mechanisms underlying cancer development and could potentially serve as targets for future cancer therapies.

### Hyper-methylated genes have interactions with diabetic and cancer-related proteins and pathways

**Fig. 3:**
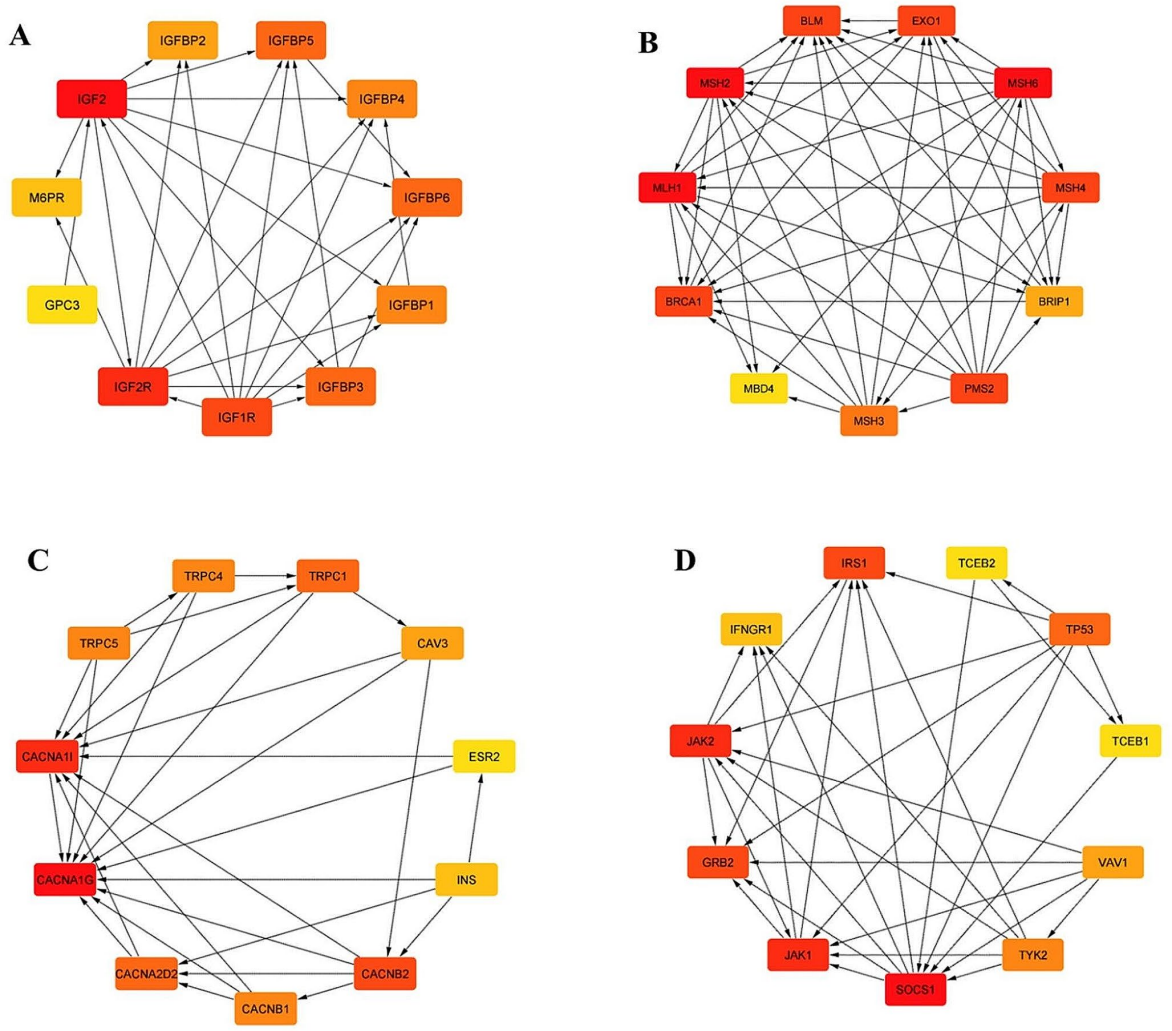
PProtein-protein interaction (PPI) network of IGF2, Socs1, MLH1, and CACNA1G with other proteins. The PPI network shows the interactions between IGF2, Socs1, MLH1, and CACNA1G, which are four downregulated genes in colorectal cancer (CRC), and other proteins. The network is divided into four clusters, each of which represents each protrin in its network. The interactions between these four proteins are complex and not fully understood

*CACNA1G*, *IGF2*, *MLH1*, and *SOCS1* proteins were analyzed using the STRING database and illustrated using Cytoscape software. The PPI network constructed for IGF2 showed that this gene could interact with more than 10 genes. Among them, *IGF2R*, *GPC3*, *IGFBP1*, *IGFBP2*, *IGFBP3*, *IGFBP4*, *IGFBP5*, and *IGFBP6*, have the highest connectivity with others (≥ 20 interactions), and they were identified as the hub genes for CRC. The PPI network constructed for SOCS1 showed that this protein is highly connected to GRB2, IRS1, JAK1, and IFNGR1. It has been also shown that CACNA1A is related to GNB1, GNAQ, RHOA, CALM1, CALM3, and CACNB2. MLH1 is connected with proteins such as MSH2, MBD4, BRCA1, and BLM (Fig. 3). The involvement and change of gene expression of *CACNA1G*, *IGF2*, *MLH1*, and *SOCS1*, in addition to causing CRC, can cause diseases such as type 2 diabetes, Fanconi’s anemia, and toxoplasmosis. Importantly, these genes play an important role in the production pathways of insulin, aldosterone, cortisol, and GnRH.

### Hyper-methylation of CACNA1G, IGF2, MLH1, and SOCS1 is associated with a decrease in the survival of patients within 150 months

**Fig. 4:**
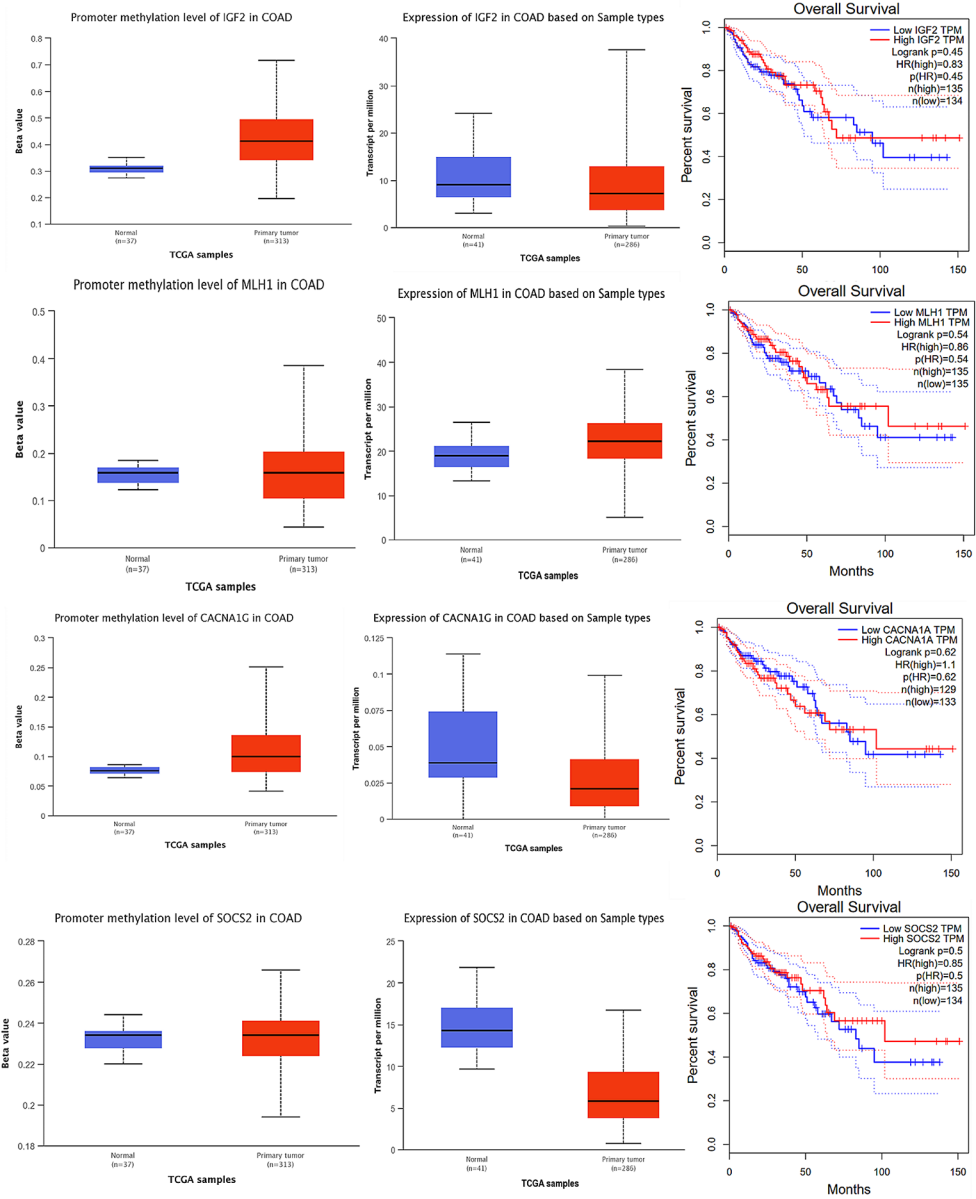
Overall expression, promoter methylation and survival analysis. Overall survival analysis of the four hub genes based on Kaplan Meier-plotter

A statistical tool called Kaplan Meier plotter was used to perform an analysis of overall survival. The promoter methylation level, expression, and survival status of four genes were checked in the UALCAN database (Fig. 4). It was found that among patients with CRC, all of these genes were hypermethylated, which means their DNA had more methyl groups attached to it than normal, and their expression was significantly decreased. It should be noted that the samples used in the overall survival analysis, which were derived from the Kaplan Meier plotter, were different from those used in the analysis of DEGs. The level of expression of hub genes, which are genes that play a central role in a network of genes, was found to be associated with the overall survival rate of CRC patients. Specifically, a poorer overall survival rate was observed in patients with higher expression levels of all hub genes. This suggests that the expression levels of these 4 genes may be useful as a prognostic marker for CRC patients.

### The change in the expression of Weisenberger panel genes initiates during the occurrence of cancer

**Fig. 5:**
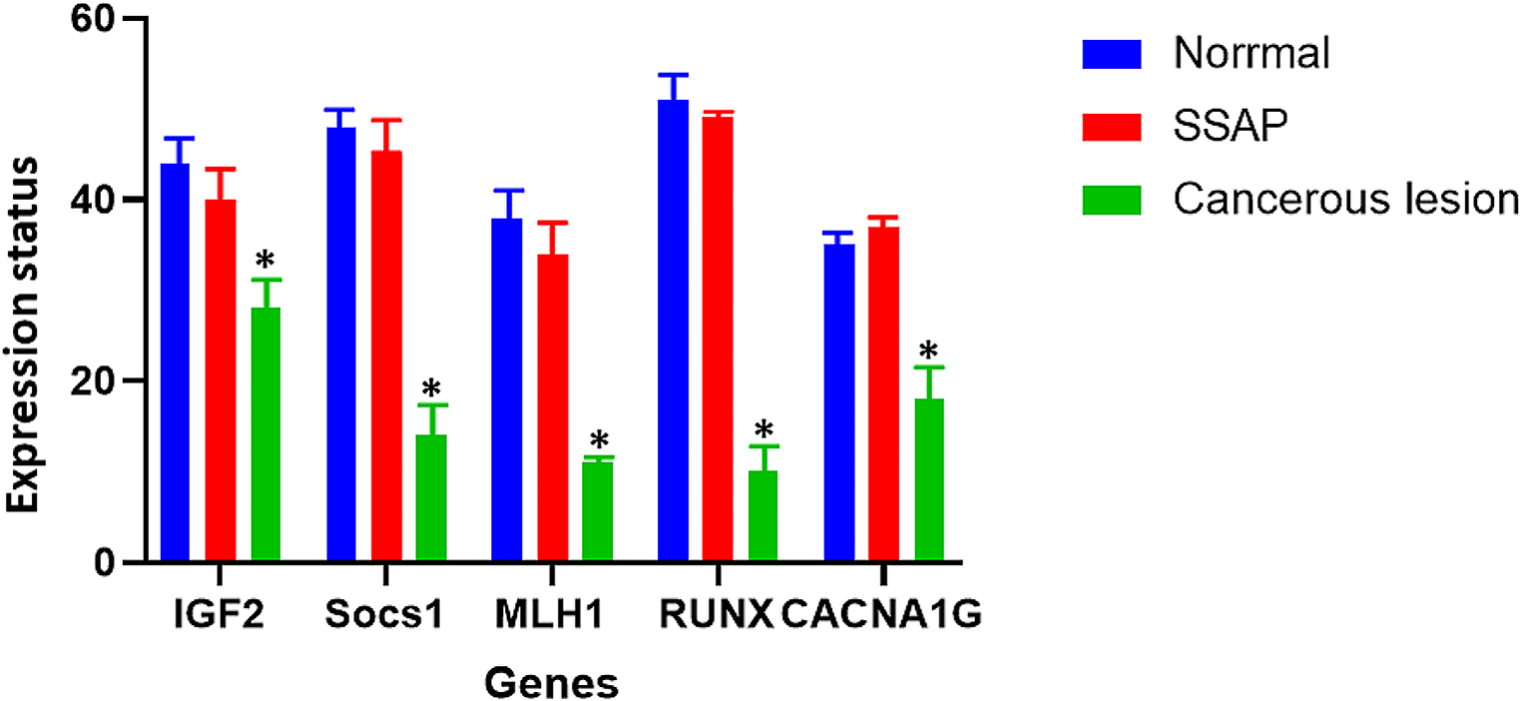
Real-time PCR analysis of gene expression levels in patient samples. The expression status of *IGF2*, *SOCS1*, *MLH1*, and *CACNA1G* genes was investigated in normal individuals, sessile serrated adenoma/polyp (SSAP), and colorectal cancer (CRC) patients. The results showed that the expression level of these genes was not changed in SSAP compared to normal individuals, but was significantly decreased in CRC patients (Interaction: *P* = 0.0006, Row Factor: *P* < 0.0001, Column Factor: *P* < 0.0001). Based on bioinformatics studies, these four genes were identified as potential prognostic markers for CRC

In this study, it has been concluded that based on bioinformatics studies of these 4 genes, they can serve as suitable prognostic markers. To understand the onset of decreased expression of these four genes in SSAP or CRC, the expression status of these genes was investigated in samples. The result of real-time PCR showed that the expression level of *IGF2*, *SOCS1*, *MLH1*, and *CACNA1G* was not changed in SSAP compared to normal individuals. However, it was significantly decreased in CRC patients (Fig. 5).

### Weissenberg panel genes are hyper-methylated in SSAP and CRC

**Fig. 6:**
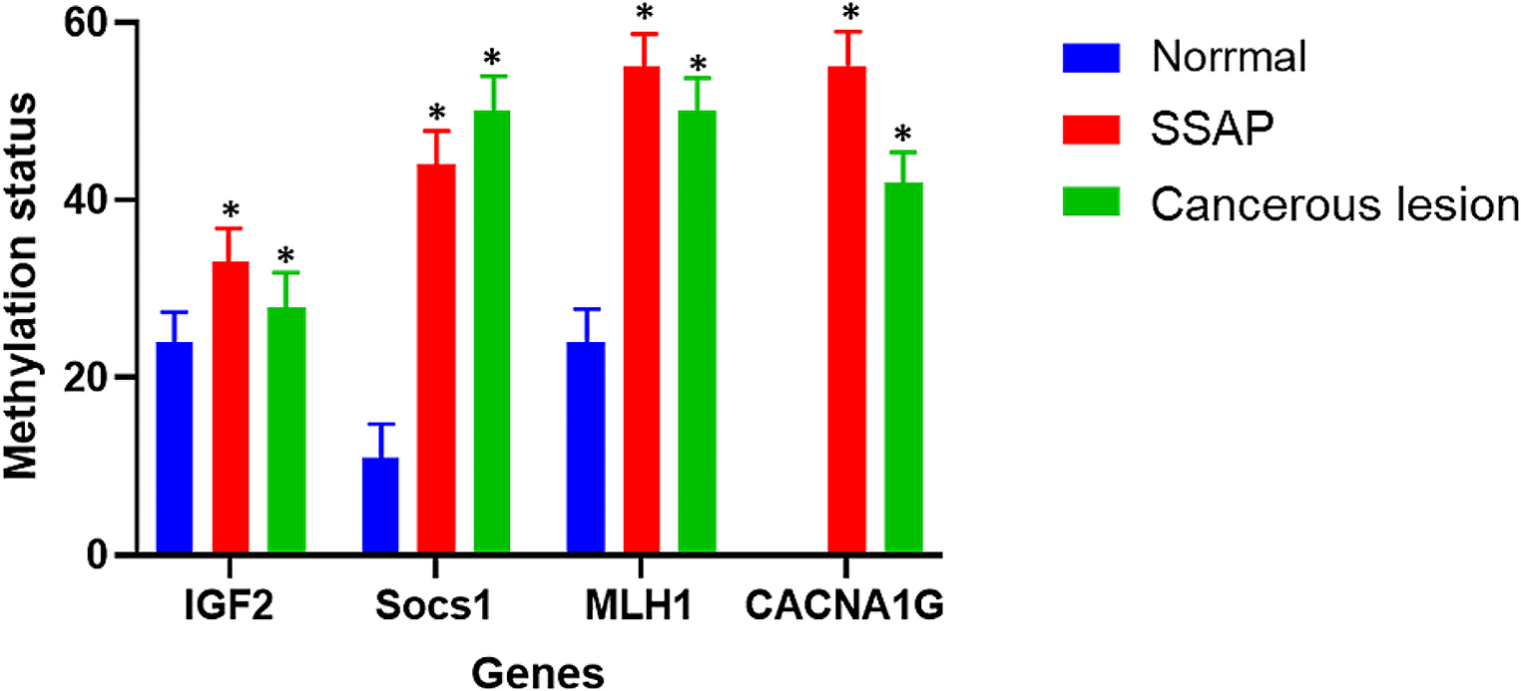
Percentage of methylation between normal, sessile serrated adenoma polyp and colorectal cancer samples. The promotor of *IGF2*, *Socs1*, *MLH1*, and *CACNA1G* was significantly hypermethylated in both SSAP and CRC groups

**Fig. 7:**
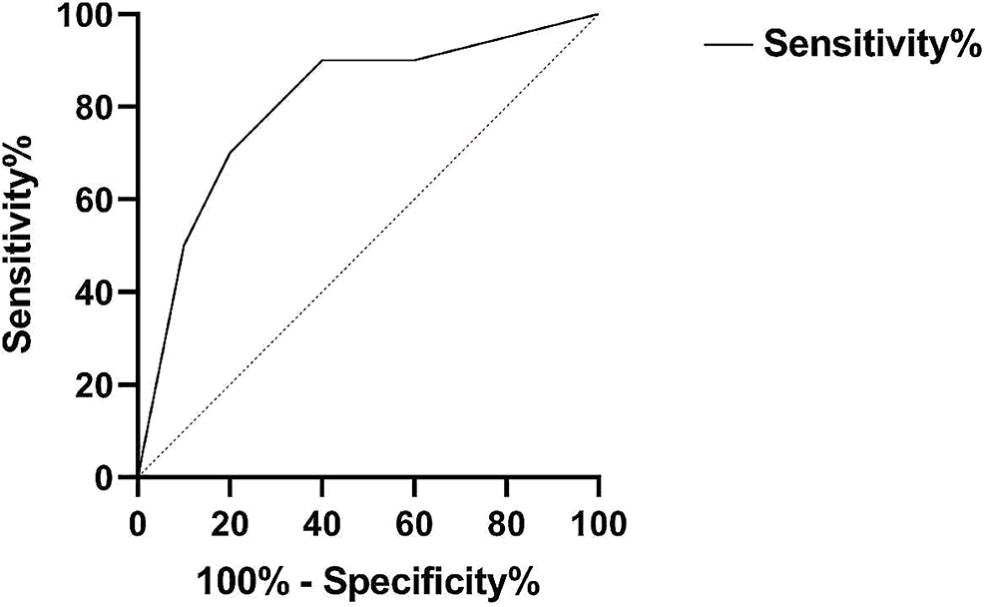
A receiver operating characteristic curve. The ROC curves of methylation status in COAD and SSAP samples

The methylation pattern was determined for 4 genes from the DNA extracted from the tissue of people with polyps and cancerous lesions (Fig. 6). The promoter of *IGF2*, *SOCS1*, *MLH1*, and *CACNA1G* genes was found to be significantly decreased in SSAP samples compared to normal samples. Additionally, it was shown that the hypermethylation condition was more severe in CRC patients compared to SSAP cases and normal individuals. The results of the ROC curve analysis showed that the AUC for *IGF2*, *SOCS1*, *MLH1*, and *CACNA1G* genes were 0.77, 0.81, 0.74, and 0.71, respectively. This suggests that these genes have moderate to good diagnostic accuracy in discriminating between CRC patients and normal individuals (Fig. 7).

## Discussion

CRC, as a major health issue in Iran, is a diagnostic challenge due to the lack of distinct symptoms. Namely, abdominal symptoms and gastrointestinal bleedings are frequently caused by non-cancerous diseases. Furthermore, the availability of resources for CRC diagnosis through the setting of colonoscopy is limited [35]. In addition, current predictive models for Suffer from poor accuracy as they Rely only on clinical symptoms. The diagnostic process for CRC is complex and involves various factors, such as the patient, the consulting physician, and the healthcare system. Understanding this process is crucial for identifying preventable factors and decreasing the impact of diagnostic delay on CRC prognosis [36]. Prior studies on CRC have revealed potential molecular and cellular mechanisms involved in the development, progression, and metastasis of this malignancy [37].

SSA polyps are precancerous lesions that can increase the likelihood of developing colon cancer in the future [38]. This study utilized various bioinformatics techniques to identify the important genes responsible for SSAP and CRC development. Recent studies suggest that bioinformatics is crucial in identifying pathways, survival, expression, and pathways associated with SSAP [39, 40]. The findings can be useful for developing precise and personalized treatments for the disease. Aberrant DNA methylation of CpG islands is widely seen in human colorectal tumors, but if this methylation occurs in promoter regions, it causes gene silencing and non-expression of an intended gene. The observed results stem from a meticulous comparison of microarray data between patients with SSAP and those with CRC against normal datasets. By employing GEO2R and the GEPIA database, we identified genes specifically expressed in CRC but not in SSAP, highlighting their potential involvement in CRC pathogenesis (Fig. 2). The ontology analysis of these genes unveiled their significance in CRC development and progression. Notably, key genes like *IGF2*, *SOCS1*, *MLH1*, and *CACNA1G*, consistent with the Weissenberg panel, emerged as down-regulated CRC-specific genes, corroborating earlier findings. To ascertain their utility as biomarkers, we assessed the expression and methylation status of these genes across SSAP, invasive adenocarcinoma, and normal tissue samples. Our findings revealed a significant decrease in gene expression levels in CRC samples compared to SSAP and normal tissue counterparts, aligning with previous studies by Komori et al. and Wentzensen et al. These results underscore the potential role of these genes in CRC pathogenesis and their viability as diagnostic and prognostic markers. In accordance with our results, in 2004, Komori et al. analyzed 8 CRC lesions and 8 normal tissue samples, they found that 1,062 genes were up-regulated and 1,004 genes were down-regulated in CRC tissues compared with normal tissue samples. Among the differentially expression genes, *CACNA1G*, *IGF2*, *MLH1*, and *SOCS1* were identified as being significantly down-regulated in CRC tissue samples. The down-regulation of these genes suggests that they may play a role in the development and progression of CRC [41]. In research conducted by Wentzensen and colleagues, 10 samples of colorectal adenoma tissue and 10 samples of normal tissue were examined. The results demonstrated that 4,322 genes were up-regulated in colorectal adenoma tissue compared to normal tissue. On the other hand, 4,374 genes were down-regulated in colorectal adenoma tissue compared to normal tissue. Among the down-regulated genes, the researchers identified *IGF2*, *SOCS1*, *MLH1*, and *CACNA1G* as being significantly decreased in colorectal adenoma tissue samples [42]. In our study, in terms of methylation analysis, *IGF2*, *SOCS1*, *MLH1*, and *CACNA1G* were hyper-methylated in both SSAP and CRC. In a study by Mun-Kar et al., it has been found that *CACNA1G* and *SOCS1* were hyper-methylated in giant colorectal polyps compared with normal colon tissues [43]. In another study, the researchers investigated the use of DNA methylation analysis as a diagnostic marker for CRC. The study analyzed the methylation status of regulatory regions of the *IGF2* gene in CRC tissues and normal colon tissues using a methylation-specific PCR approach. The study found that the methylation status of *IGF2* regulatory regions could be used as a diagnostic marker for CRC, with high sensitivity and specificity. These findings suggest that DNA methylation analysis can provide valuable information for the early detection and diagnosis of CRC [44]. Since hyper-methylation occurs in both SSAP and CRC samples, suitable markers for distinguishing between colon cancer may not be available. Our study suggests that for the diagnosis of colon cancer, it is better to examine the expression status rather than the methylation status of *IGF2*, *SOCS1*, *MLH1*, and *CACNA1G* genes. This is because the expression of these genes occurs specifically downregulated in cancer, not in SSA polyps. According to Fig. 5, the decreased expression of *IGF2*, *SOCS1*, *MLH1*, and *CACNA1G* is also observed in type 2 diabetes and Fanconi anemia. In a study, the researchers compared the expression of *SOCS1* in insulin-resistant human subjects and controls. They found that the expression of *SOCS1* was significantly decreased in insulin-resistant subjects, suggesting a potential role for *SOCS1* in the development of insulin resistance and type 2 diabetes [45]. The mechanism behind the observed phenomenon could involve common regulatory pathways governing gene expression and DNA methylation in both SSAP and CRC. One potential mechanism is the dysregulation of epigenetic machinery, such as DNA methyltransferases, leading to aberrant DNA methylation patterns shared between SSAP and CRC. This dysregulation may occur due to mutations or alterations in upstream signaling pathways involved in epigenetic regulation, such as the Wnt signaling pathway, which is frequently dysregulated in CRC. Additionally, shared environmental factors or microenvironmental cues within the colonic epithelium may contribute to similar epigenetic changes in both SSAP and CRC.

The researchers also analyzed genetic variants in the *CACNA1G* gene in a large cohort of Chinese subjects with and without type 2 diabetes. They found that several genetic variants in the *CACNA1G* gene were significantly associated with an increased risk of type 2 diabetes, suggesting a potential role for *CACNA1G* in the development of the disease [30].

## Conclusion

In conclusion, our study sheds light on the molecular landscape underlying CRC and SSAP, providing valuable insights into their pathogenesis and potential diagnostic and therapeutic implications. By leveraging bioinformatics techniques, we identified key genes implicated in CRC development, including *IGF2*, *SOCS1*, *MLH1*, and *CACNA1G*, which exhibited significant downregulation in CRC tissue samples compared to SSAP and normal tissues. These findings corroborate earlier studies and underscore the potential utility of these genes as diagnostic and prognostic biomarkers for CRC. Importantly, our results suggest that assessing the expression status of these genes may be more informative for CRC diagnosis than examining their methylation status, as hypermethylation was observed in both SSAP and CRC samples. The observed downregulation of these genes in CRC aligns with their involvement in other diseases like type 2 diabetes and Fanconi anemia, suggesting potential common molecular mechanisms underlying these conditions. Moving forward, further validation of these findings in larger cohorts and diverse populations is warranted to establish their clinical utility. Integration of these genes into existing diagnostic algorithms or prognostic models could enhance the accuracy of CRC detection and prognostication, ultimately improving patient outcomes. Moreover, exploring the therapeutic potential of targeting these genes or their associated pathways may pave the way for personalized treatment strategies for CRC patients. Future research endeavors should focus on elucidating the precise molecular mechanisms governing the dysregulation of these genes in CRC and exploring their therapeutic implications in preclinical and clinical settings. Additionally, investigating the interplay between genetic and environmental factors in modulating the expression of these genes could provide further insights into CRC pathogenesis and identify novel targets for intervention.

## Data Availability

The data presented in this study are available on request from the corresponding authors.

## Abbreviations

CRC: Colorectal cancer
CIMP: CpG island methylator phenotype
SSAP: Serrated sessile adenomas/polyps
CIN: characteristics are chromosomal instability
MSI: Microsatellite instability
DNMTs: DNA methyltransferases
NBI: Narrow-band imaging
PcG: Polycomb group
APC: Adenomatous polyposis coli
BRCA1: Breast cancer 1
CDH1: Cadherin 1
CDKN2A: Cyclin-dependent kinase inhibitor 2 A
MLH1: MutL homolog 1
PTEN: Phosphatase and tensin homolog
TP53: Tumor protein p53
VHL: Von Hippel-Lindau tumor suppressor
GEO: Gene Expression Omnibus database
GEPIA: Gene Expression Profiling Interactive Analysis
BP: Biological processes
MF: Molecular function
KEGG: Kyoto Encyclopedia of Genes and Genomes
DAVID: Database for Annotation Visualization and Integrated Discovery
CC: Cellular components
CACNA1G: Calcium Voltage-Gated Channel Subunit Alpha1 G
IGF2: Insulin-like Growth Factor 2
SOCS1: Suppressor of Cytokine Signaling 1
